# Diagnosis

**Published:** 1880-05

**Authors:** B. F. Coy

**Affiliations:** Baltimore


ARTICLE III.

### DIAGNOSIS: AS LEARNED IN OUR PROFESSIONAL COLLEGES.

BY B. F. COY, D. D. S., BALTIMORE.

"Pathology is the shady side of physiology." This quotation from Allbutt most naturally leads us to conclude that there is a dividing line between health and disease; and, at the same time, brings us to the thought that this same intangible division is the point that has eluded the scientific explorer in medicine in all its branches, leaving the novice in diagnostics in the slough of despond; and often, yea, very often, baffled the most experienced, the most careful and thoughtful searcher after the mysteries of disease—the departures from normal or healthy conditions.

The very pinnacle of health leaves us but one short step to that *shady side*—a departure from normality; and taken unawares, our brakes, from disuse, self-confidence and complacency, are unprepared for the emergency. Acute disorders have done their mischief before assistance can be utilized and therapeutics are powerless to give their aid.

Medical science, including all departments connected with the healing principle, or the art and science of restoration to normality, offers so boundless a field for speculation and discovery that the newly fledged are given frequently to look upon their puny wings as what the world has so long stood in need of, and the glory of whose powers and flight must astonish as well as greatly benefit the suffering specimens of this planet; and, perhaps, in some wondrous manner only dreamed of, cast their scintillating lights to other and less fortunate spheres.

'Tis very true that most of us are to learn our best remembered lessons from our mistakes and our overdrawn theories, and in maturer years to compensate the human race by the acquisition of a wisdom that in enthusiastic youth was not ours; although of book-knowledge we seemed possessed of all worth knowing.

How few of the medical fraternity can look back with complacence upon his early experience at the bedside, when some blind, deeply-hidden case was presented for a deliberate or hasty diagnosis. Where then were his theories—his book-lessons or his repeated lectures? And where that confidence he was so sure was to sustain him? How seemed to him, then, the old fogy whose slow-spoken words, cautiously-given opinions, and modest manner but deep thought, whose gray locks and declining years were but the evidence of many a hard fought battle in solving the mysteries of disease and assisting nature to recover again her throne and power?

A faulty diagnosis as to the lesion or disease, its true extent and the degree of departure from the standard of health, will lead to error as to the amount of assistance needed, as well as to the nature of that assistance.

It matters little in what department of the healing art, whether in any of the so-called specialties or in those approaching more nearly that position that entitles them to be regarded as a profession, the same tendency to speculation and premature gushing becomes the bane of the newly fledged enthusiast, and only time, study and hard rubs will sift and utilize the wild theories and overweening confidence natural to the novice and the new disciple.

We find our mills, or colleges, so-called, of the medical and dental professions turning loose upon all communities young men possessed, in their own estimation, of far superior attainments to what can be accorded them by those not immediately interested in the issue of their diplomas. This is one of the misfortunes of our time; and until we can bring our schools to a graduated course of lectures and instruction, or, in other words, advance each lecture course to meet the requirements of our students, we are teaching *primer* lessons at each course; and, consequently, turn out a class of primaries instead of educated gentlemen. 'Tis no design of this article to nominate a curriculum for colleges, either in medicine or dentistry, but simply to endeavor to impress the fact that we are not properly educating men to become diagnosticians in their professions to that degree to meet the wants of the age or the common needs of humanity, in their earlier practice or, in many cases, experiments.

Must graduates of our schools always go blundering along in diagnosis and misapplied treatment until, by a long series of dangerous or fatal experiments, they have acquired that wisdom and foresight their parchment says they possess?

These remarks apply not only to medicine and dentistry proper, but to all specialties under either. We are speaking whereof we know, with an experience of five years as lecturer in an important chair in a dental college; still we had to contend against a curriculum that gave no opportunity for an advance course.

We have some evidence that thinking men of both professions are becoming awakened to the necessity of some method by which graduates shall be submitted to greater discipline, and given the chance to gather more knowledge to enable them to be better qualified to unravel the mysteries of disease before beginning their experiments to learn whether there is disease or otherwise.

